# Supplementary material for: Antimicrobial Peptides Designed against the Ω-Loop of Class A β-Lactamases to Potentiate the Efficacy of β-Lactam Antibiotics
Source: Antibiotics (Basel). 2023 Mar 10;12(3):553. doi: 10.3390/antibiotics12030553 (PMC10044640; doi:10.3390/antibiotics12030553)

Supplementary Data

Synthesis and Characterisation of peptides designed against the omega loop of class A β-lactamases to reverse antimicrobial resistance

Sarmistha Biswal[1,2], Karina Caetano[2], Diamond Jain[1], Anusha Sarrila[1], Tulika Munshi[2ϒ], Rachael Dickman[3,4], Alethea B. Tabor [3], Surya Narayan Rath[5], Sanjib Bhakta[2]*, Anindya S. Ghosh[1]*

Bacterial culture conditions for biological assays

Liquid cultures of *M. tuberculosis* and *M. smegmatis* were grown in Middlebrook (MB) 7H9 medium supplemented with 10% (vol/vol) albumin-dextrose-catalase (ADC; Difco, MD), 0.05% (vol/vol) Tween 80, and glycerol. Solid growth was on Middlebrook 7H10 agar plus 10% (v/v) oleic acid-ADC (OADC, Difco). Liquid cultures of *E. coli, K. pneumonia,* and *S aureus* were grown in Luria-Bertani (LB broth or LB agar), and Cation Adjusted Mueller Hinton (MHB) was used for MIC studies in *E. coli, K. pneumonia,* and *S aureus* strains. All the bacterial cultures were grown at 37^0C and 180rpm conditions with appropriate antibiotics at the following concentration viz., kanamycin at 50 µg/mL, chloramphenicol at 20 µg/mL, and tetracycline at 25 µg/mL.

Solid-Phase Peptide Synthesis

Automated synthesis protocol

Prior to synthesis, the resin (H-His (Trt)-2-ClTrt resin, loading 0.71 mmol g^{-1}, or Fmoc-Arg(Pbf)-Wang resin, loading 0.59 mmol g^{-1}, 100 mg per peptide) was swollen in DMF (3 mL) for 15 min. Peptides were synthesised simultaneously by automated Fmoc SPPS on a Biotage Syro I automated parallel peptide synthesiser, in 5 mL PP reaction syringes with a frit. During all reaction and washing steps the resin is vortexed for 20 s at 1 minute intervals. Following each step, the reagents are removed by vacuum filtration. The total reaction volume in each step was 1.5 mL. All reagents were dissolved in DMF, excluding DIPEA which was dissolved in NMP. Fmoc deprotection protocol: A solution of piperidine in DMF (40% v/v, 1.5 mL) was added to the syringe. After 3 min this was removed by vacuum filtration and a second portion of piperidine in DMF added (20% v/v, 1.5 mL). After 10 min this was removed by vacuum filtration and the resin washed with DMF (6 x 1.5 mL).

Coupling protocol: Each amino acid was double coupled to ensure complete reaction. The Fmoc amino acid (4 eq), HATU (4 eq) and DIPEA (8 eq) were added to the syringe. After 40 min the reagents were removed by vacuum filtration. Fresh solutions of the Fmoc amino acid (4 eq), HATU (4 eq) and DIPEA (8 eq) were then added to the syringe for a second time and reacted for a further 40 min. The reagents were removed by vacuum filtration and the resin washed with DMF (4 x 1.5 mL). Fmoc amino acids used were: Fmoc-Glu(O*t*Bu)-OH, Fmoc-His(Trt)-OH, Fmoc-Ile-OH, Fmoc-Leu-OH, Fmoc-Arg(Pbf)-OH, Fmoc-Ser(*t*Bu)-OH, Fmoc-Thr(*t*Bu)-OH, Fmoc-Tyr(*t*Bu)-OH.

Cleavage and Salt Exchange

Following the final Fmoc deprotection, the final washing and cleavage steps were performed manually in the syringe, using a syringe cap and plunger. The resin was continually agitated throughout the cleavage step by shaking at 480 rpm on an IKA KS130 basic platform shaker. Before cleavage, the resin was washed with CH_2Cl_2 (3 x 2 mL), MeOH (3 x 2 mL) and ether (3 x 2 mL) and dried in vacuo for 30 min. The cleavage cocktail (TFA: H2O:TIPS, 37:1:2 v/v/v, 2 mL total) was then added to the resin. This was agitated continuously for 3 h before evacuating directly into a 15 mL Falcon tube containing cold ether (7 mL). The frit was washed with TFA (200 µL) which was also added to the Falcon tube. The volume was then made up to 14 mL with more cold ether. This was centrifuged at 4000 rpm at 5 °C for 15 min. The ether was poured off the resulting pellet and fresh ether added to resuspend the pellet, before a further round of centrifugation (4000 rpm, 5 °C, 10 min). The resuspension and centrifugation process was repeated once more. The resultant pellet was dissolved in water (3 mL) and lyophilised to yield the crude peptide. Peptides requiring purification were subsequently dissolved in water and filtered through a 0.45 µm syringe filter before HPLC. The resulting peptide TFA salts were treated with HCl to produce the HCl salts. The peptides were dissolved in 10 mM HCl (7 mL) and left to stand at rt for 10 min, followed by lyophilisation. The peptides were then redissolved in 10 mM HCl, left to stand, and lyophilised again. This dissolution and lyophilisation process was repeated once more, then the peptides were dissolved in water (5 mL) and lyophilised to yield the final products.

DMF was HPLC grade and was used as purchased from Fisher Scientific. Ether refers to diethyl ether. Solvent used for HPLC was all HPLC-grade and used directly from the bottle. Standard Fmoc amino acids and resins were used as purchased from Merck. An Eppendorf centrifuge model 5810R was used for centrifugation of peptide products before freeze drying by a SP Scientific VirTis BenchTop Pro. Peptides were purified by preparative reverse phase

HPLC on an Agilent Infinity 1260 Prep system with an Infinity II fraction collector, using an Agilent ZORBAX 300SB-C18 7 µm 21.2 x 250 mm column and detection at 214 nm. A linear solvent gradient of 25 to 40% MeCN (0.1% TFA) in H_2O (0.1% TFA) over 7.5 min was used, at a flow rate of 20 mL min^{-1}. Analytical HPLC traces were collected on an Agilent Technologies 1260 Infinity system using a Dr Maisch GmbH Reprosil Gold 200 C8 5µm 250 x 4.6 mm column, with detection at 214 nm. For His peptides, a linear solvent gradient of 5 to 95% MeCN in H_2O (0.1% TFA) over 30 min was used, at a flow rate of 1 mL min^{-1}. For Arg peptides, a linear solvent gradient of 5 to 95% MeCN in H_2O (0.1% TFA) over 10 min was used at a flow rate of 1 mL min^{-1}. Accurate mass spectra were recorded on an Agilent LC system connected to an Agilent 6510 Q TOF mass spectrometer.

Construction of MD simulation Study

The molecular structures of peptides were initially improved and prepared for simulation using the software Discovery Studio 3.5. (Accelrys Software Inc). MD simulation was performed using the AMBER99SB-ILDN force field of the GROMACS 5.0.4 package. AMBER force field is generally used for an amino acids-based synthesized peptide which contains mostly C, N, O, and H atoms. Each peptide was in a protonated state when the PH was set to its default value. The TIP3P force field was used to build the water model for salvation which was embedded in a dodecahedron box with 10 Å of minimum edge distance. An electrical neutral state was achieved for all six systems by adding appropriate NA or CL ions with the replacement of water molecules After that, the steepest descent energy reduction was applied to each system until a force tolerance of 100 KJ/mol was reached. Following, systems were equilibrated at 300K (NVT) for 100ps backbone restraints, followed by 100 ps of pressure equilibrium (NPT). All of the restraints were removed during NPT. At the time of temperature coupling, the time constant (τT) was set for 0.1 ps, whereas the isotropic Parrinello-Rahman barostat of 1.0 bar was used during pressure coupling with a time constant (τT) of 2.0 ps. Long-range Coulomb interactions were handled using the particle mesh Ewald approach. Restriction of all bond angles was conducted for 2fs time step using linear constraint solver (LINCS) algorithm. For coulomb interactions and van der Waals forces, cut-off distances were kept at 1 nm. Finally, MD simulations of all of these six systems were performed independently for a time period of 100ns time scale.

Table S1: Physical and chemical parameters of peptides.

**Peptide Sequences**	**EYRIR** (P1)	**TYRLR** (P2)	**TSHLR** (P3)	**TTHIR** (P4)	**ETHIH** (P5)	**TSHLH** (P6)	**ESRLH** (P7)	**ESHIH** (P8)	**ESRIH** (P9)	**TYHLH** (P10)
**Molecular weight (g/mol)**	735.84	707.83	612.69	626.71	635.68	593.62	640.70	621.65	640.70	669.74
**Isoelectric Point**	9.9	11.2	11.1	11.1	6.0	8.0	7.8	6.0	7.8	7.9
**Net charge at pH 7.0**	-0.8	2.0	1.1	1.1	1.0	0.2	0.1	-0.8	0.1	0.2
**Average hydrophilicity**	1.0	0.3	0.1	0.0	0.0	-0.6	0.8	0.1	0.8	-1.1

Table S2: MIC of ampicillin against *M. tuberculosis* with peptides through HT-SPOTi.

Strain	MIC value Antibiotics(mg/L)
	**Amp**
MTB H37Rv	**125**
MTB H37Rv + P1	>250
MTB H37Rv + P2	>250
MTB H37Rv + P3	>250
MTB H37Rv + P4	>250
MTB H37Rv + P5	16
MTB H37Rv + P6	4
MTB H37Rv + P7	8
MTB H37Rv + P8	8
MTB H37Rv + P9	ND
MTB H37Rv+ P10	ND
DMSO	>250
Isoniazid	2

Footnotes: Amp: Ampicillin; ND: not determined

Table S3: MIC of various bacteria with peptides in absence of β-lactam antibiotics.

Peptides	Bacteria strains		
	E. coli	***M. smegmatis***	***K. pneumoniae***
P1	128	>500	>250
P2	128	>500	>250
P3	128	>500	>250
P4	64	>500	>250
P5	128	>500	>250
P6	256	>500	>250
P7	64	>500	>250
P8	128	>500	>250
P9	128	>500	>250
P10	256	>500	>250

Figure S1. Peptide synthesis Data sheet

H-Glu-Tyr-Arg-Ile-Arg-OH HCl salt (peptide 1)

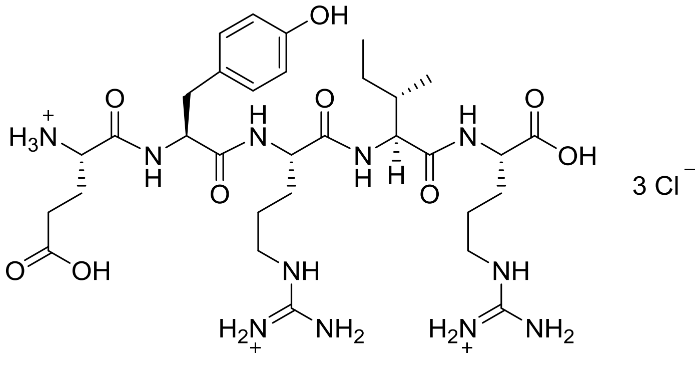

Yield: 0.3 mg, 0.41 μmol, 1%.

Accurate mass:

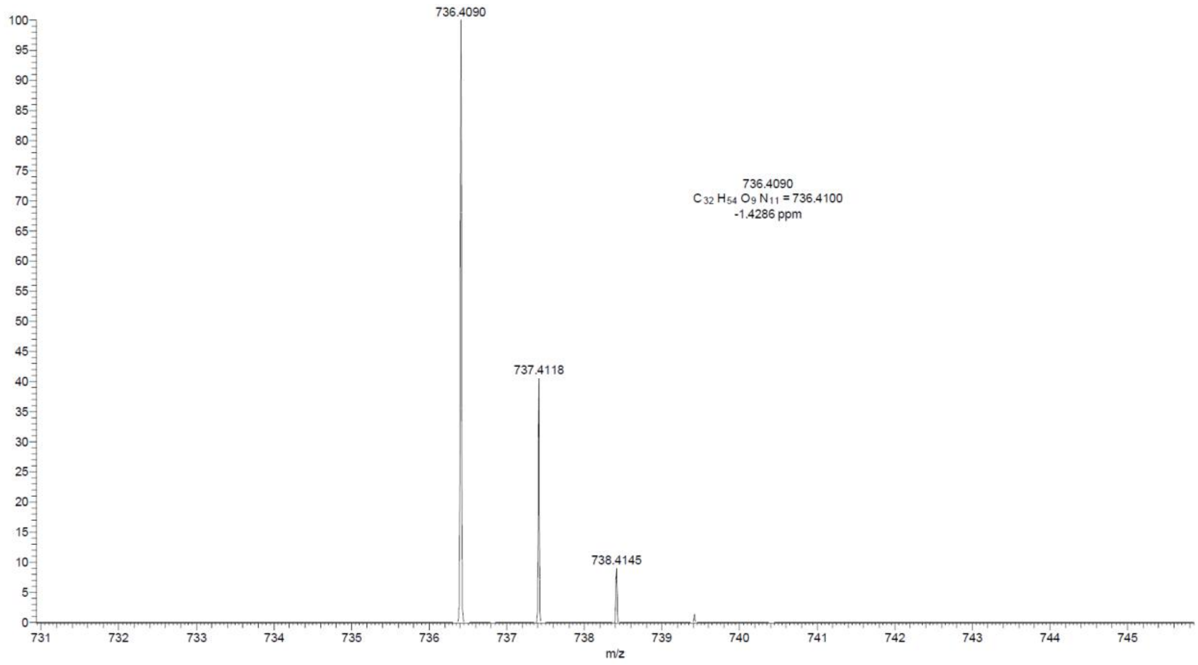

Analytical HPLC: purity 90%, retention time 5.66 min.

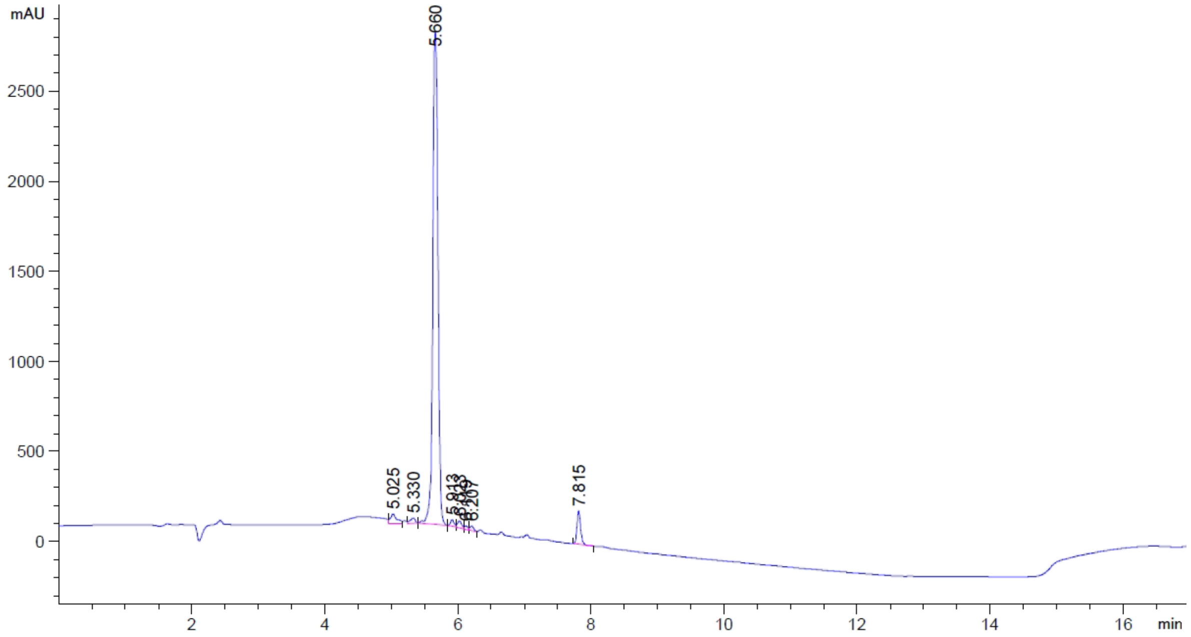

H-Thr-Tyr-Arg-Leu-Arg-OH HCl salt (peptide 2)

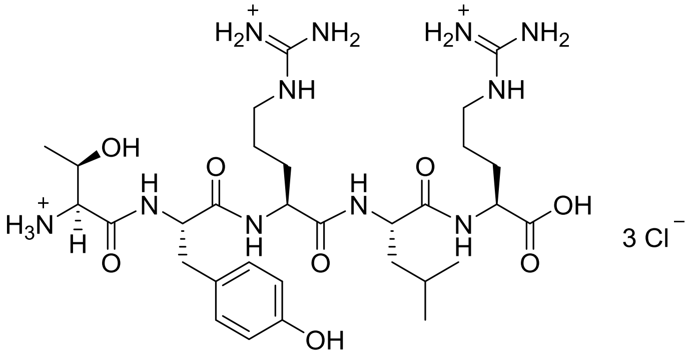

Yield: 7.3 mg, 10.3 µmol, 18%.

Accurate mass:

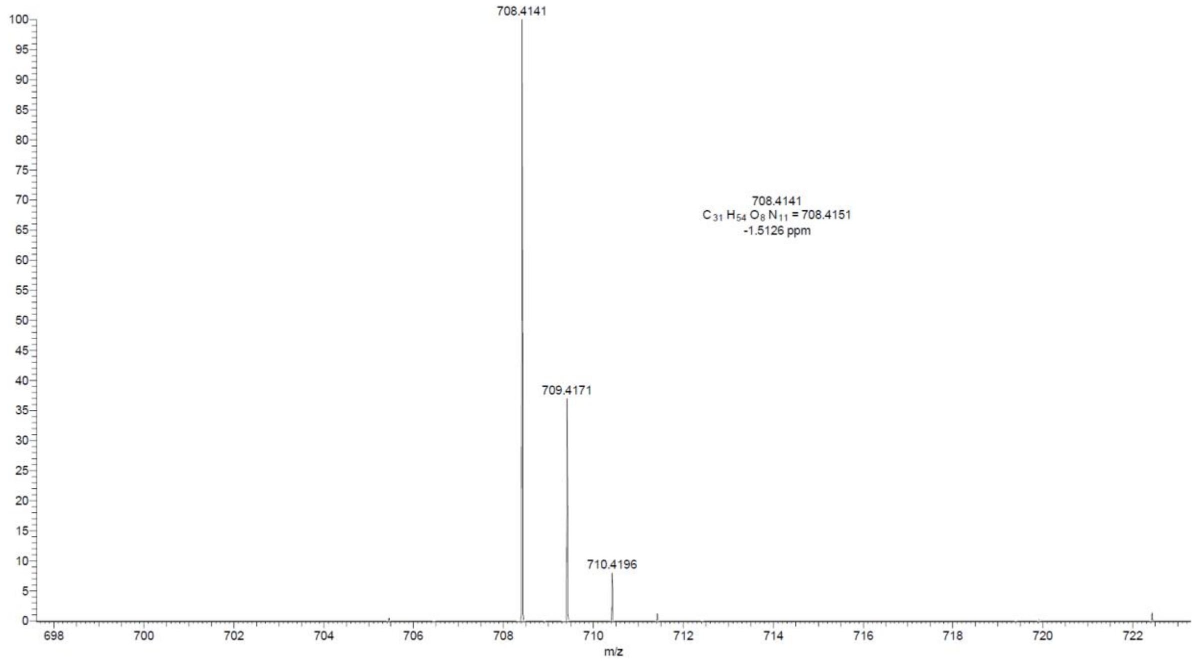

Analytical HPLC: purity 82%, retention time 5.69 min.

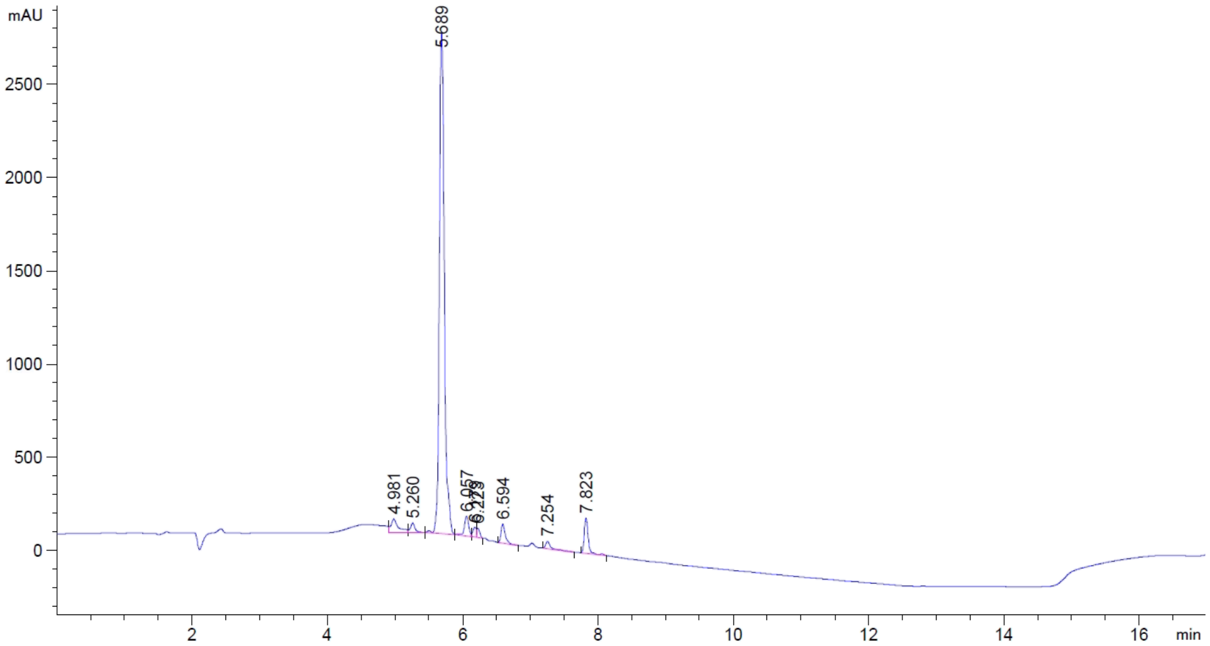

H-Thr-Ser-His-Leu-Arg-OH HCl salt (peptide 3)

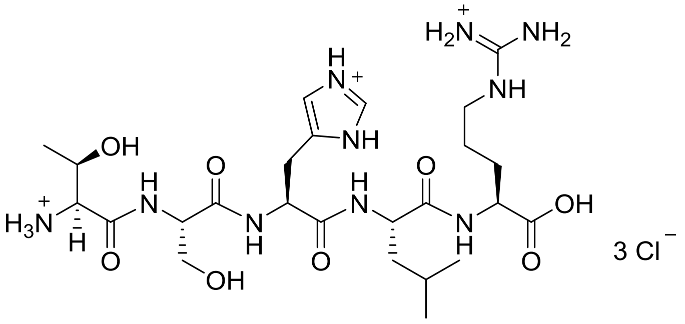

Yield: 10.9 mg, 17.8 µmol, 30%.

Accurate mass:

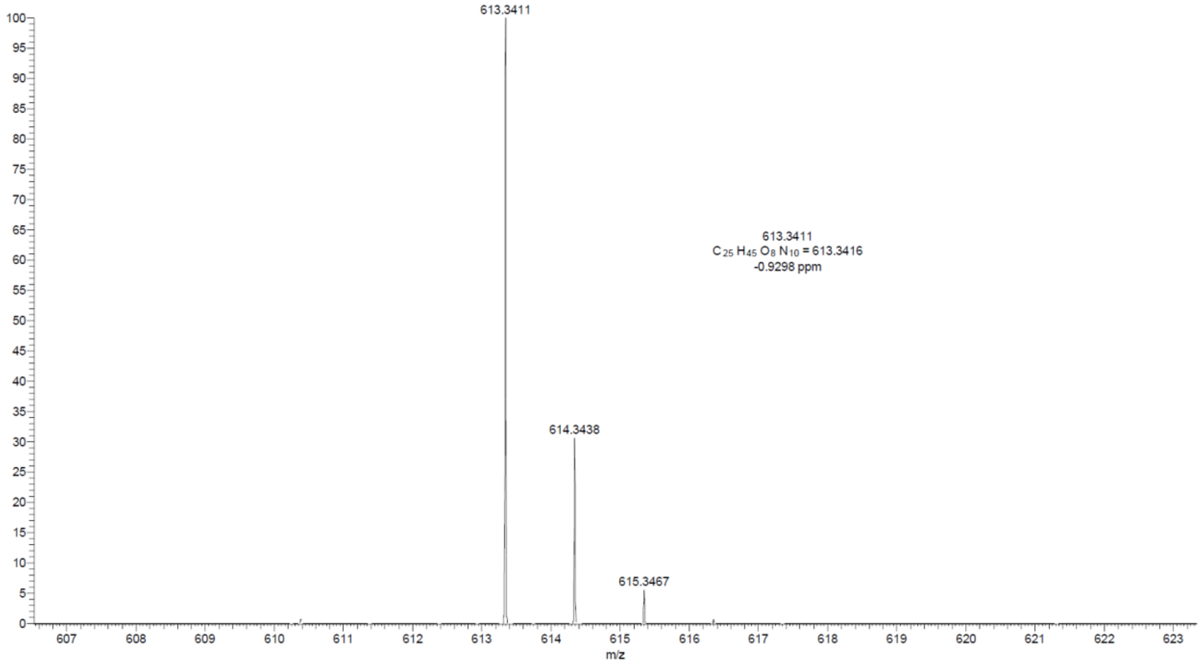

Analytical HPLC: purity 93%, retention time 5.15 min.

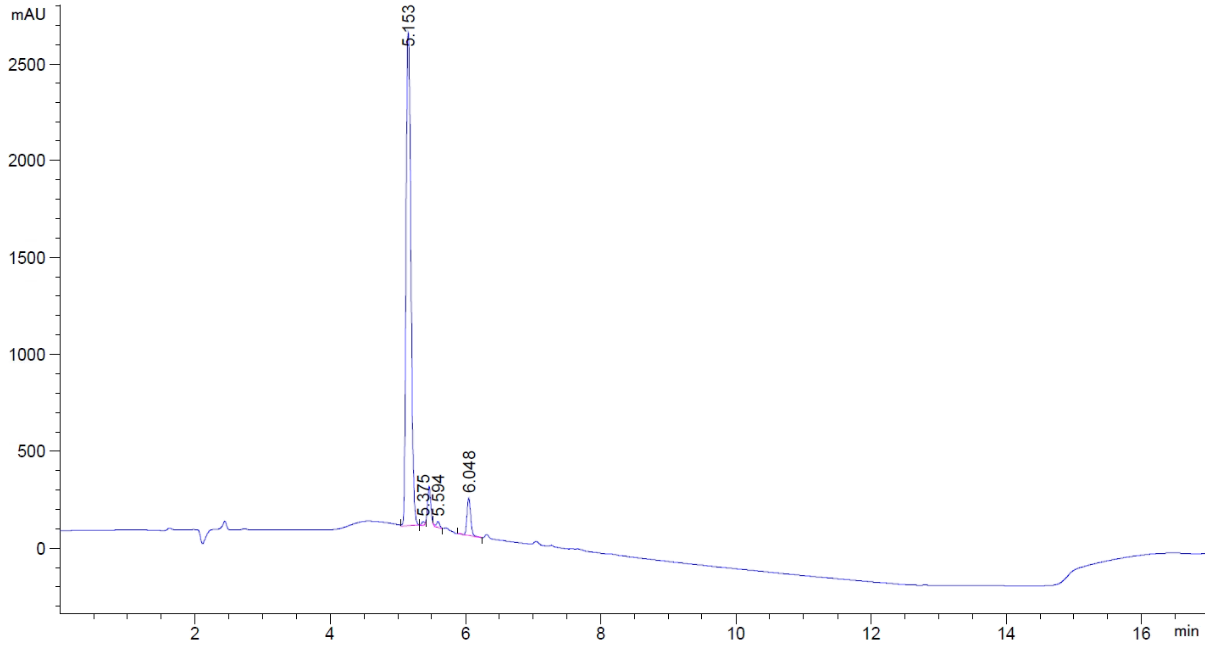

H-Thr-Thr-His-Ile-Arg-OH HCl salt (peptide 4)

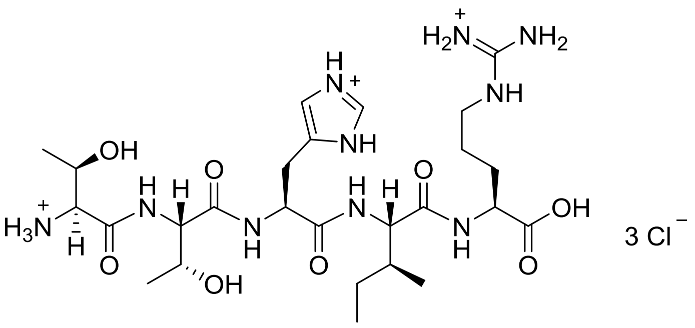

Yield: 10.4 mg, 16.6 μmol, 28%.

Accurate mass:

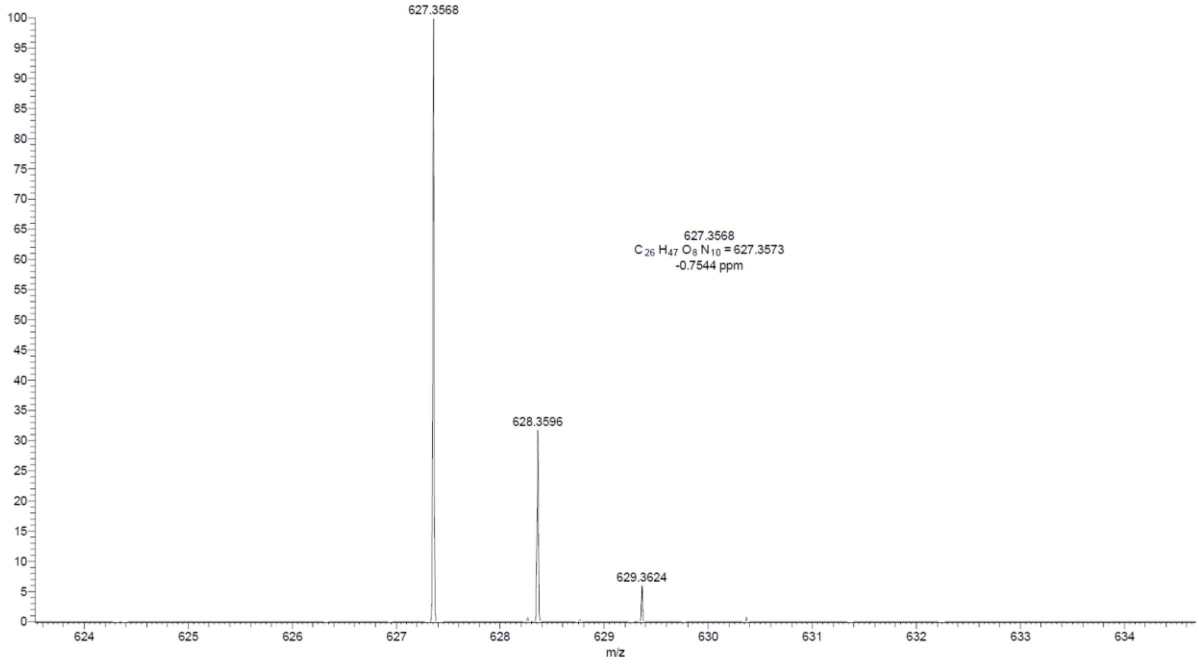

Analytical HPLC: purity 82%, retention time 5.18 min.

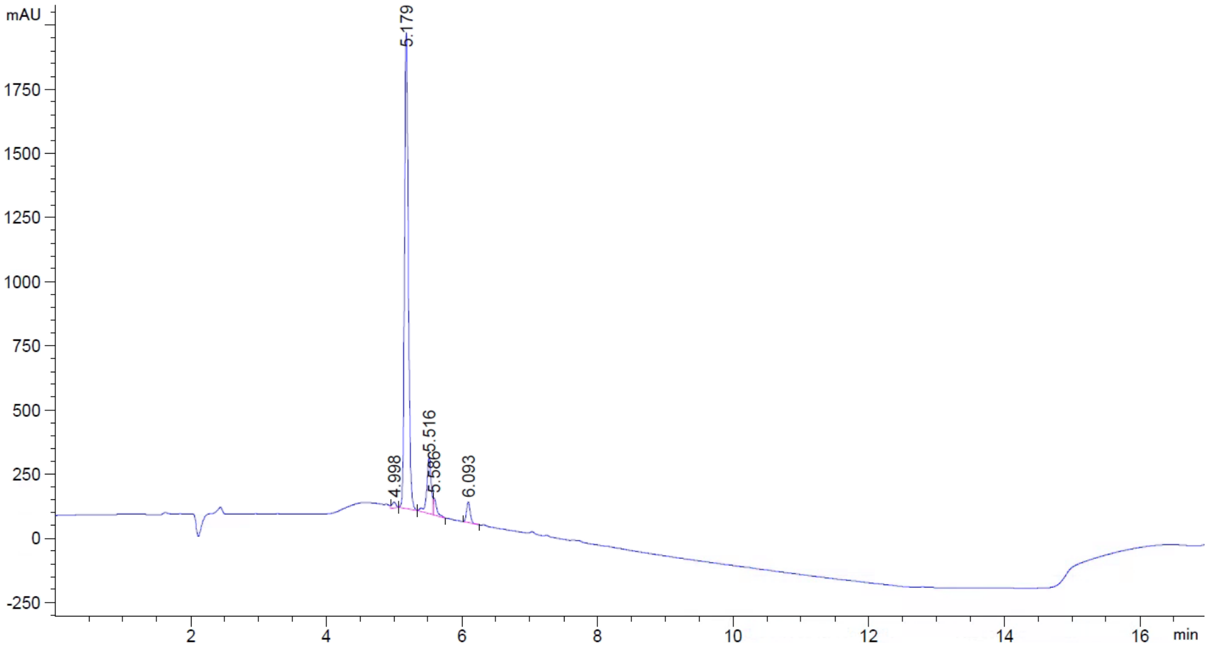

H-Glu-Thr-His-Ile-His-OH HCl salt (peptide 5)

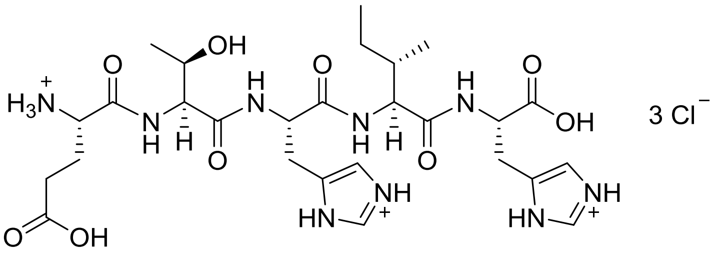

Yield: 41.2 mg, 55.4 µmol, 78%.

Accurate mass:

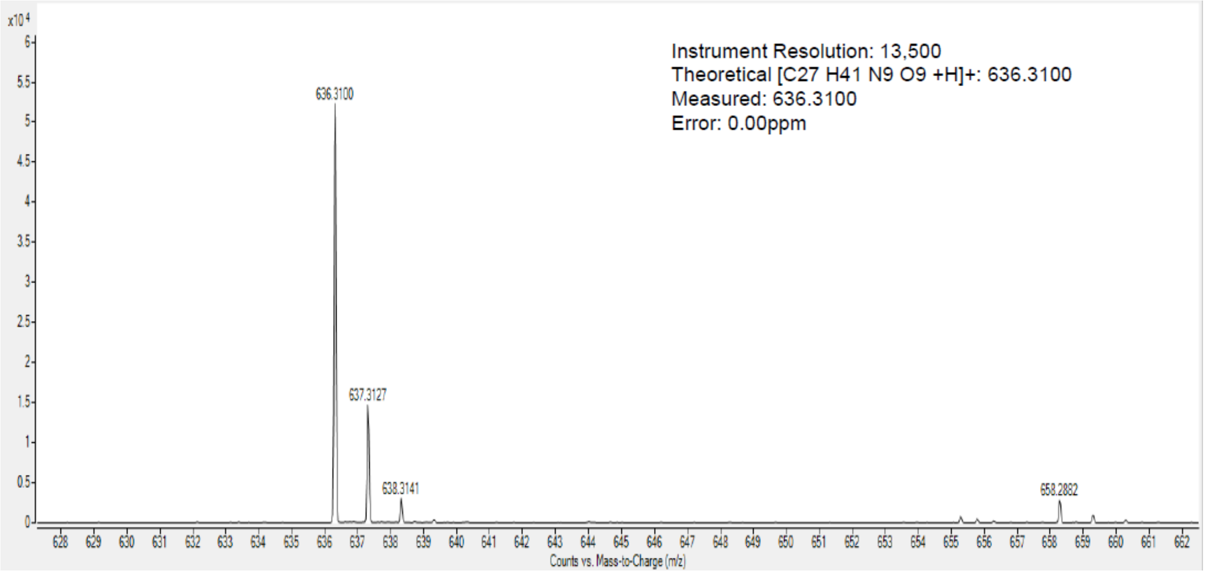

Analytical HPLC: purity >95%, retention time 8.48 min.

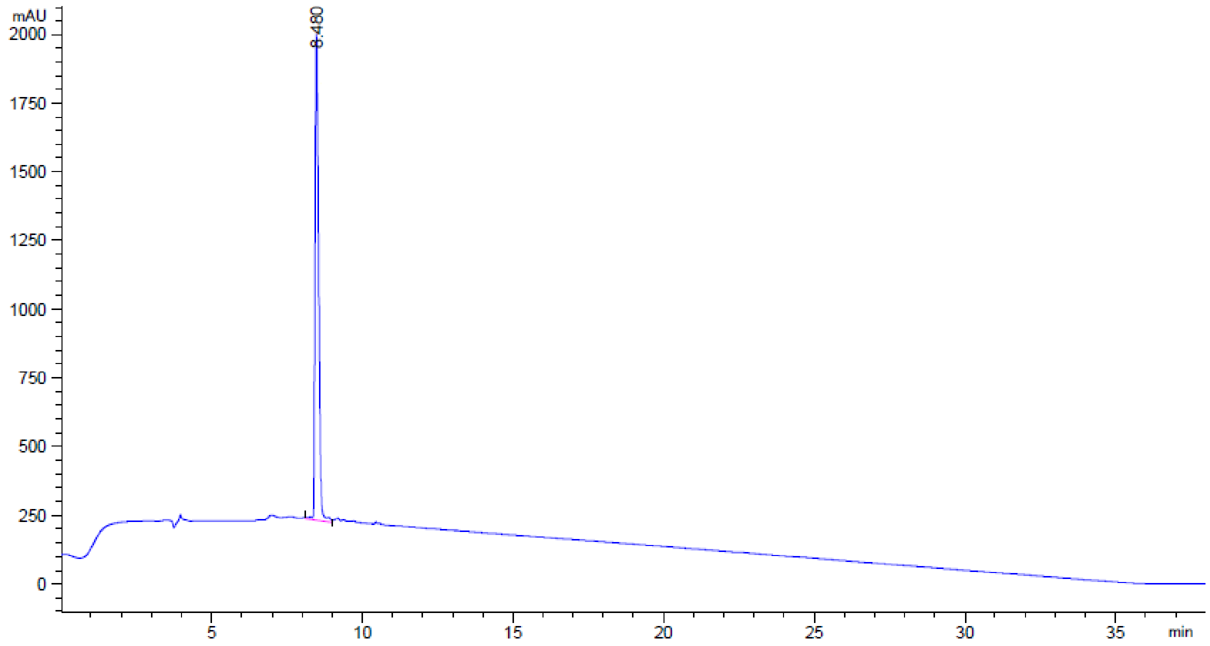

H-Thr-Ser-His-Leu-His-OH HCl salt (peptide 6)

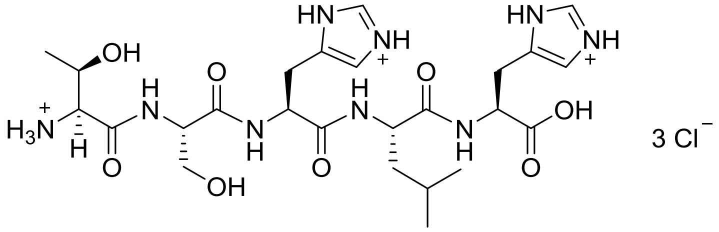

Purified by prep HPLC.

Yield: 14.1 mg, 201.1 µmol, 28%.

Accurate mass:

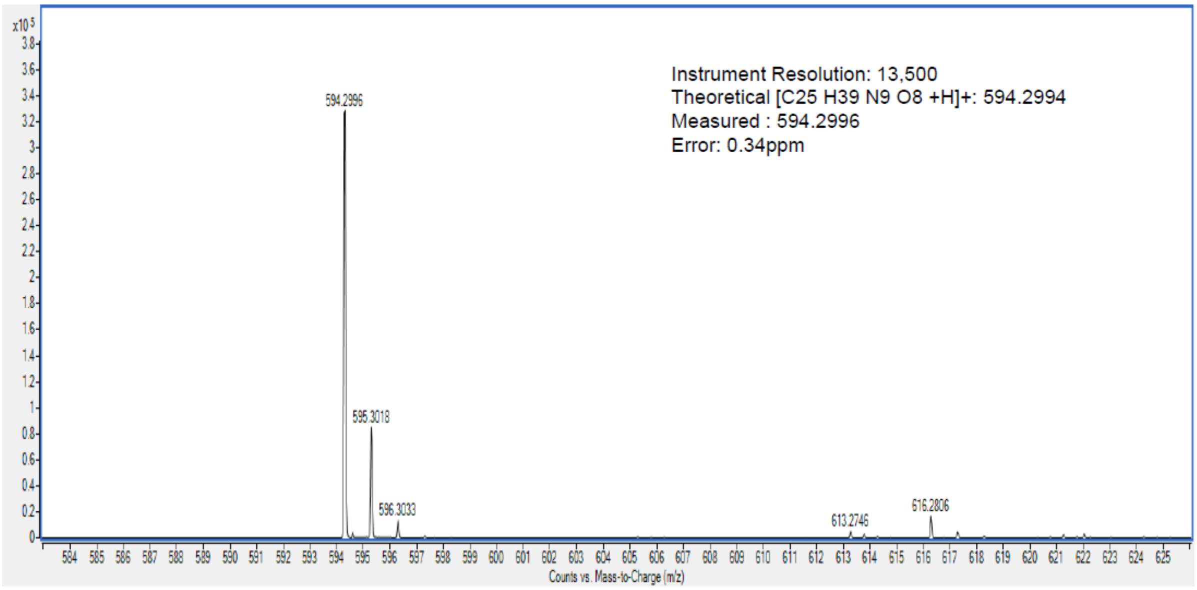

Analytical HPLC: purity >95%, retention time 8.42 min.

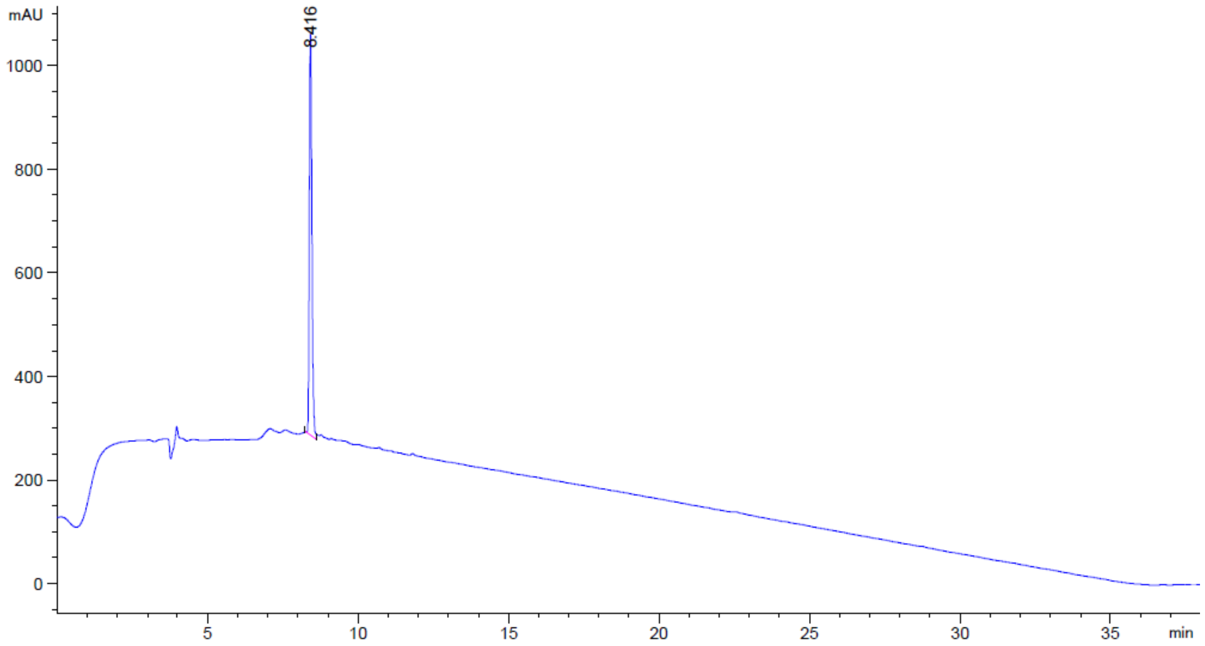

H-Glu-Ser-Arg-Leu-His-OH HCl salt (peptide 7)

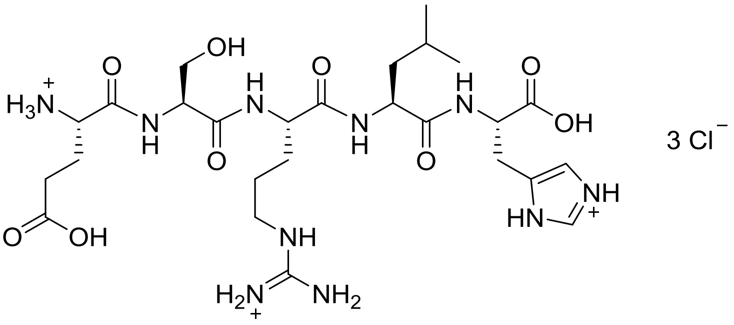

Yield: 47.4 mg, 63.3 µmol, 89%.

Accurate mass:

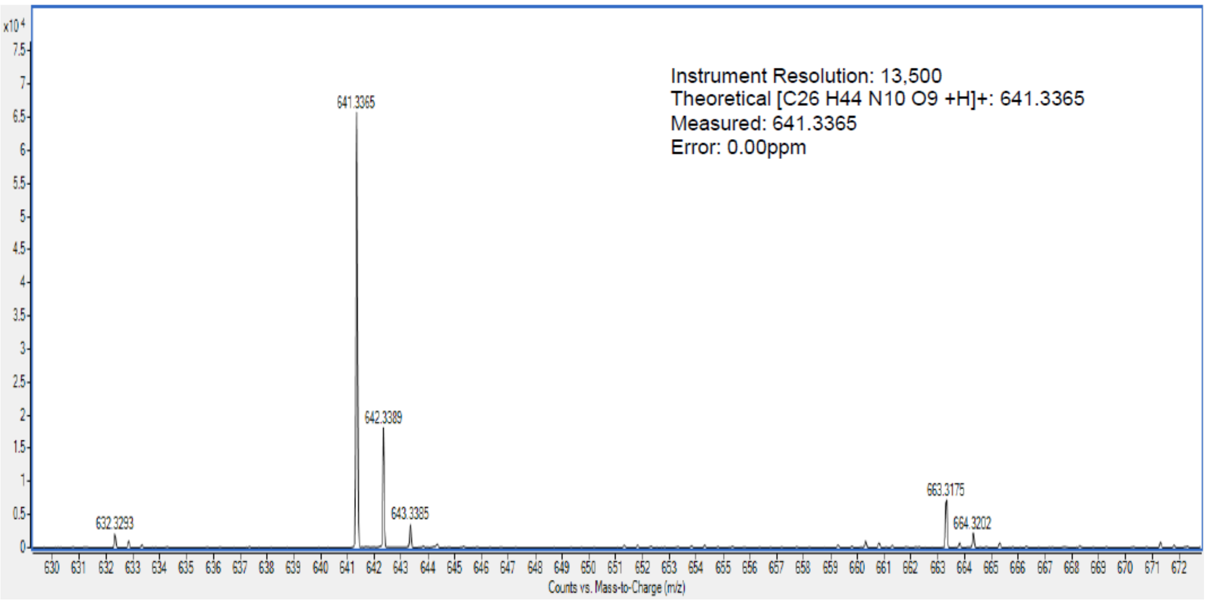

Analytical HPLC: purity >95%, retention time 8.62 min.

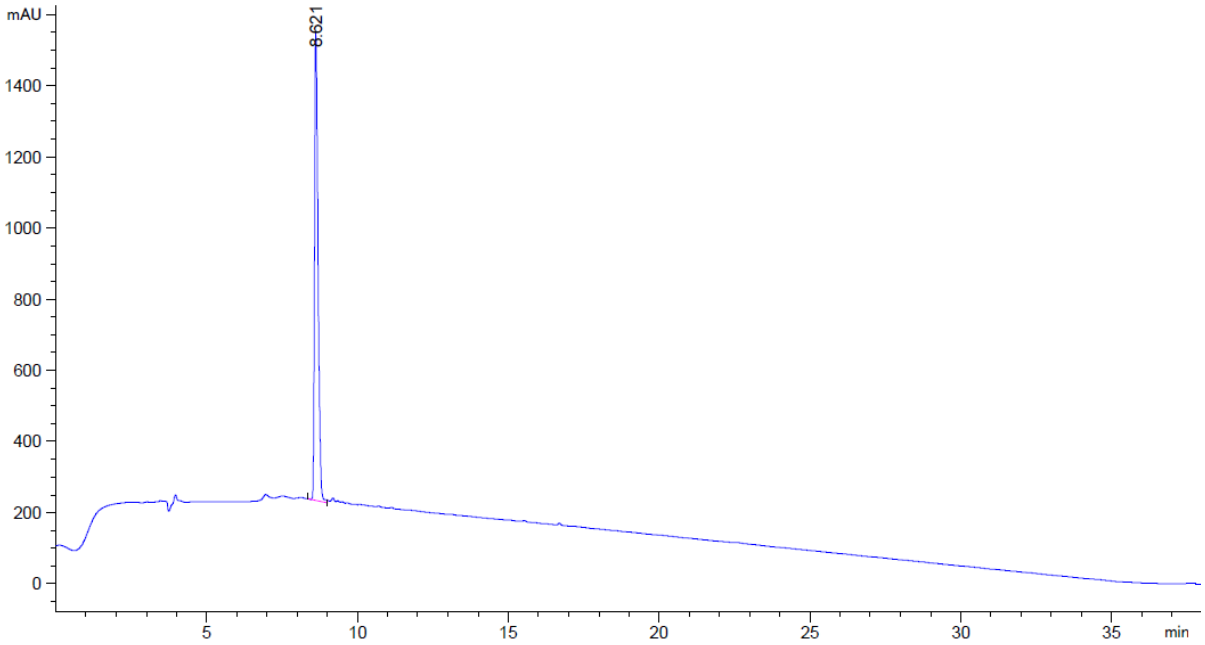

H-Glu-Ser-His-Ile-His-OH HCl salt (peptide 8)

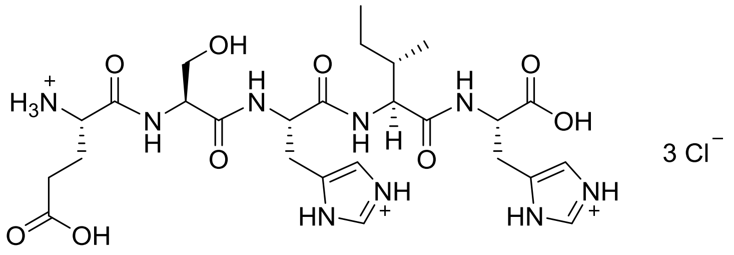

Yield: 44.3 mg, 60.8 µmol, 86%.

Accurate mass:

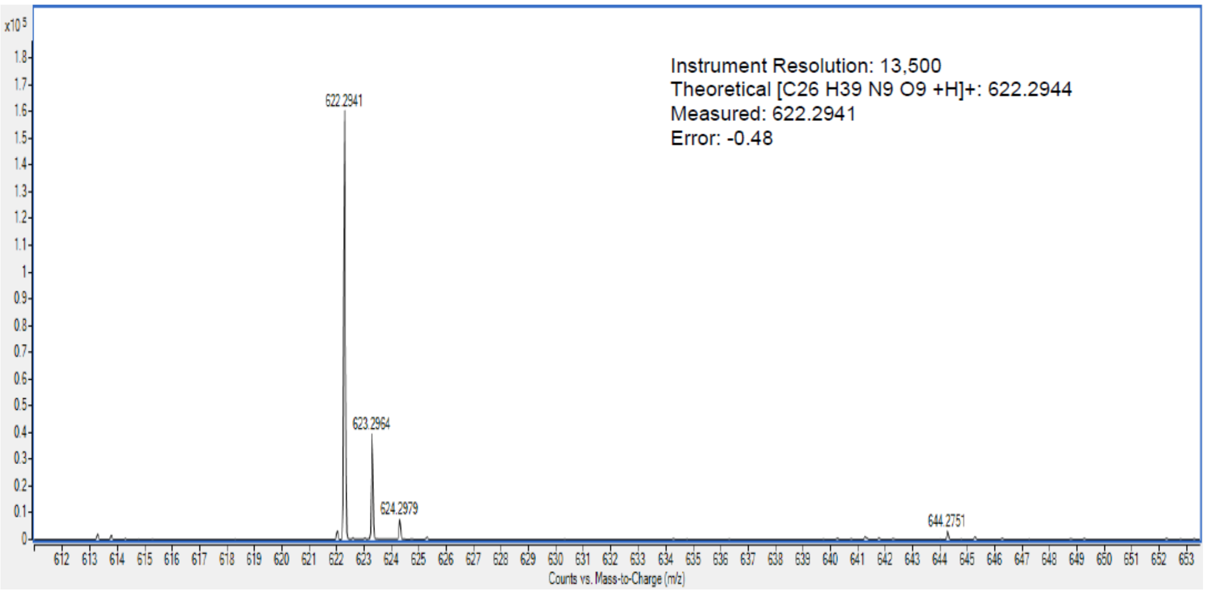

Analytical HPLC: purity >95%, retention time 7.93 min.

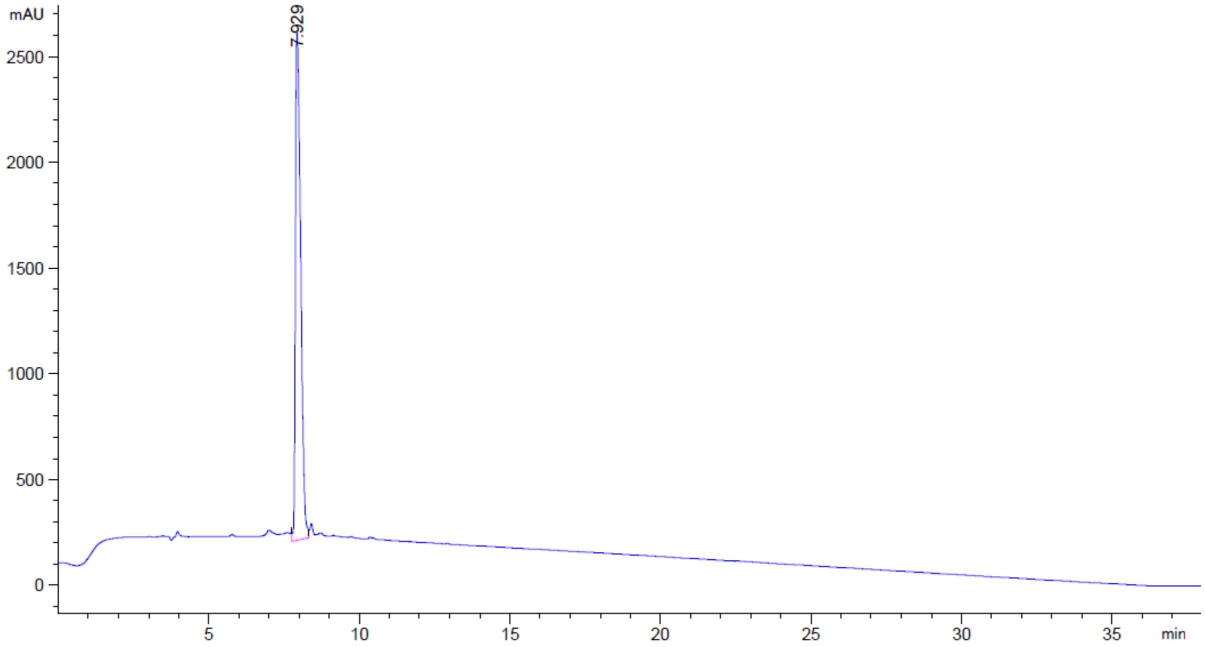

H-Glu-Ser-Arg-Ile-His-OH HCl salt (peptide 9)

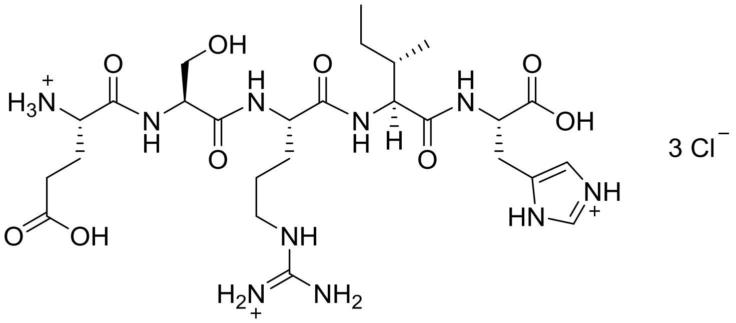

Yield: 45.7 mg, 61.1 µmol, 86%.

Accurate mass:

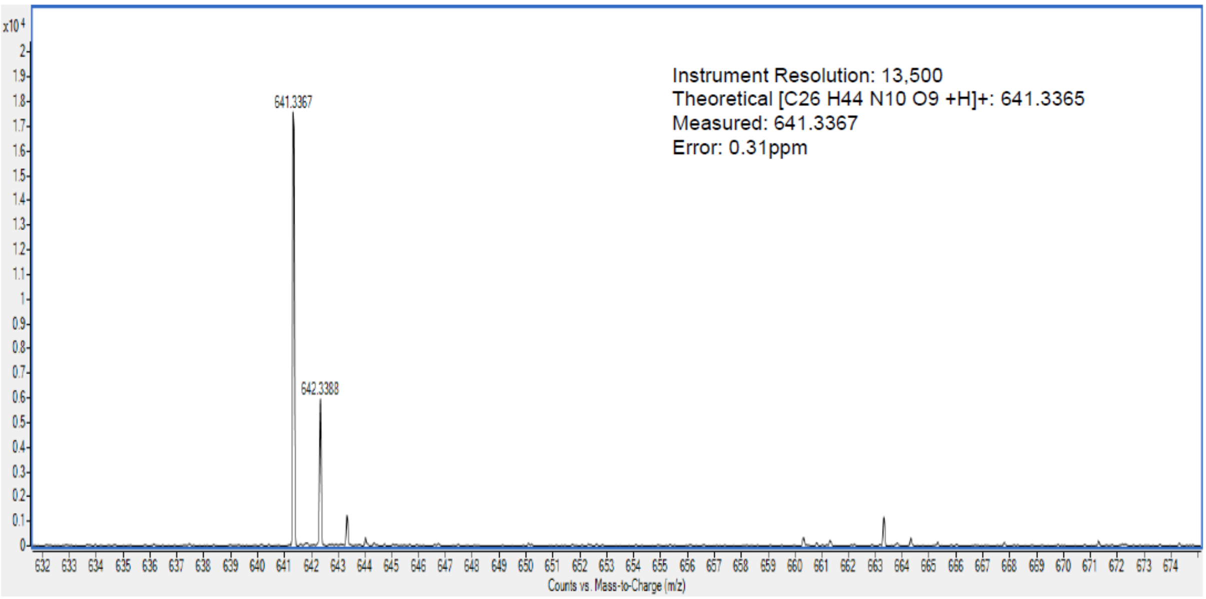

Analytical HPLC: purity >95%, retention time 8.48 min.

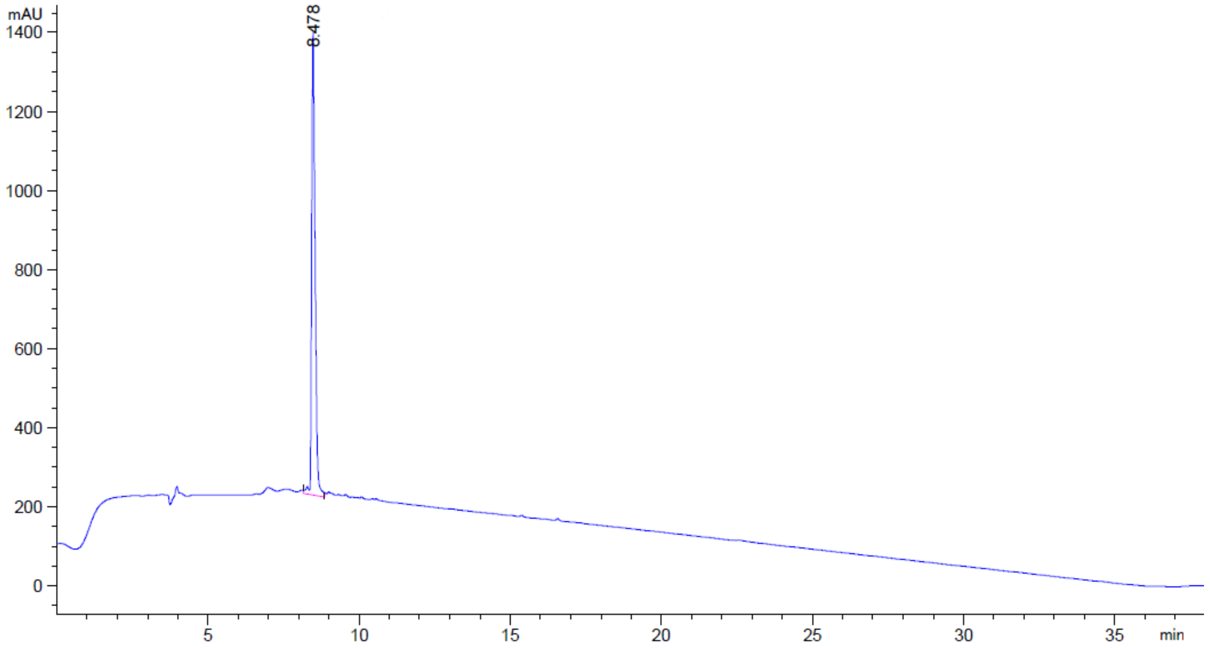

H-Thr-Tyr-His-Leu-His-OH HCl salt (peptide 10)

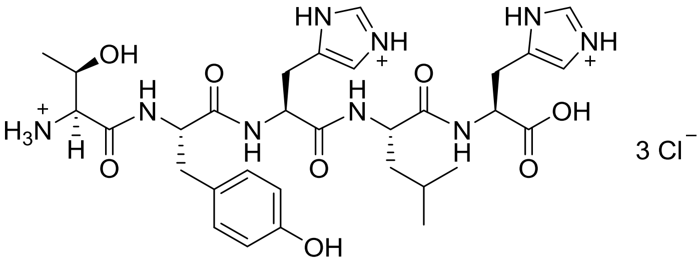

Purified by prep HPLC.

Yield: 26.8 mg, 35.5 µmol, 49%.

Accurate mass:

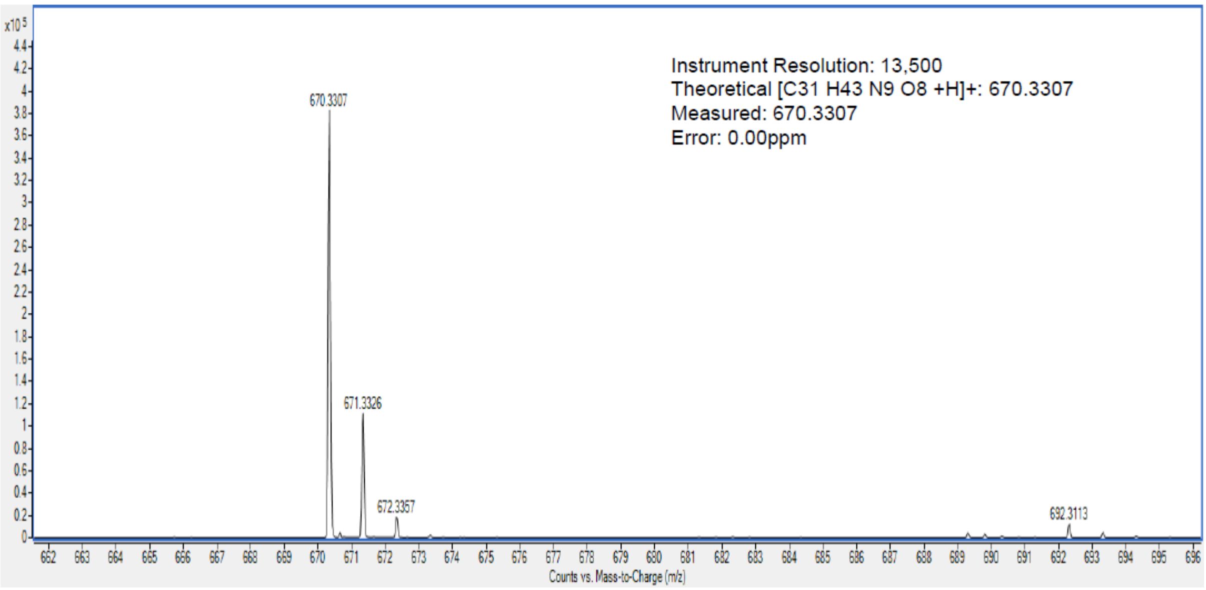

Analytical HPLC: purity >95%, retention time 10.64 min.

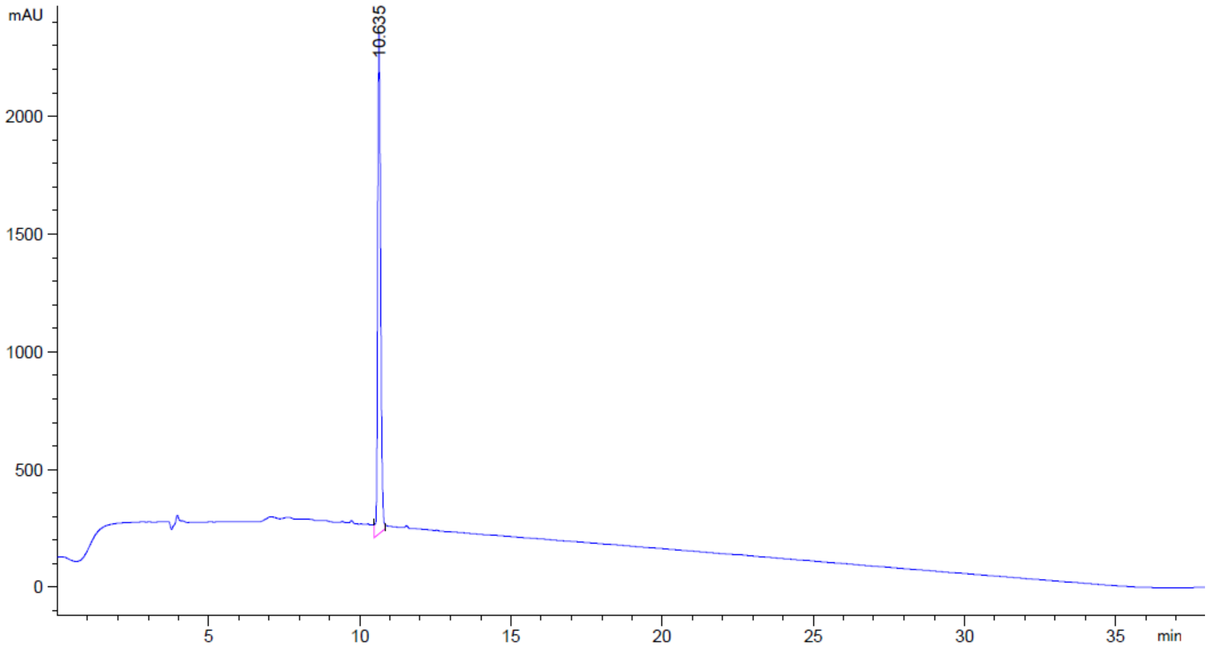

Supplement: Supplementary file 1 [file antibiotics-12-00553-s001.zip › antibiotics-2213191-supplementary.pdf]
